# Effect of the Processing Parameters on the Fabrication of MgAl_2_O_4_ Foams

**DOI:** 10.3390/ma14205945

**Published:** 2021-10-10

**Authors:** Reynaldo Morales-Hernández, Víctor H. López-Morelos, Diana Cholico-González, Francisco Fernando Curiel-López, Marco Arturo García-Rentería, Lazaro Abdiel Falcón-Franco, Victor Hugo Martínez-Landeros

**Affiliations:** 1 Instituto de Investigación en Metalurgia y Materiales, Universidad Michoacana de San Nicolás de Hidalgo, Francisco J. Mújica S/N, Ciudad Universitaria, Edificio U, Morelia 58030, Mexico; reymo_18@hotmail.com (R.M.-H.); diana.cholico@umich.mx (D.C.-G.); francisco.curiel@umich.mx (F.F.C.-L.); 2 Facultad de Metalurgia, Universidad Autónoma de Coahuila, Carretera 57 Km 5, Monclova 25710, Mexico; marcogarciarenteria@uadec.edu.mx (M.A.G.-R.); lazarofalcon@uadec.edu.mx (L.A.F.-F.); vmartinezlanderos@uadec.edu.mx (V.H.M.-L.)

**Keywords:** foams, suspensions, MgAl_2_O_4_, soap-nut extract, saponin

## Abstract

Stable MgAl_2_O_4_ foams (7–21 vol.%) were manufactured using a natural extract from the pericarp of the soap-nut fruit, saponin being the main component, as the foaming agent. The soap-nut extract is soluble in water, biodegradable, non-toxic, and has similar properties to commercial tensoactives. The stability and characteristics of the porous structure of the ceramic foams were evaluated in terms of the amount of foaming agent, content of MgAl_2_O_4_ particles, time and speed of stirring of the slurry, type of agitator, and drying temperature. It was found that the foaming capacity decreased with the percent of foaming agent and ceramic, whereas the time and speed of stirring enhanced the foamability. Foaming trials showed that stirring aqueous slurries with 3 wt.% of soap-nut extract for 2 min at 1070 or 2120 rpm, depending on the type of agitator, produced stable MgAl_2_O_4_ foams when drying at 60 °C. The mechanism of foaming is discussed. The foams were sintered at 1400 °C for 1 h under an Ar atmosphere. Observation of the sintered foam structures in the scanning electron microscope revealed nearly spherical cells with very good interconnectivity and strength to be manipulated, making them suitable as preforms for manufacturing Al-based composites by pressureless infiltration.

## 1. Introduction

Macroporous ceramics or ceramic foams are of great interest for various applications including filters of molten metals and gases at high temperatures, catalyst supports, light structural materials, biomaterials, and as preforms to fabricate near-net-shape interconnected metal matrix composites [1,2]. These types of materials are sought after due to their specific properties conferred by the low density, low thermal conductivity, large specific surface area, and permeability [2]. The characteristics of the ceramic foams concerning morphology, porosity, and pore size distribution are dictated by the processing route and thereby determine the potential applications of these materials [2]. Nevertheless, the manufacture of these macroporous structures is a technological challenge, owing to the thermodynamic instability of these systems [3]. There are different manufacturing techniques to produce ceramic foams, including replica templates, sacrificial fugitives, and direct foaming [4]. The latter is a technique of particular interest due to its simplicity, low cost, and versatility, which enables us to obtain open macroporous structures in the range of 45% to 93% in porosity [3,5]. This method involves the entrapment of a gaseous phase into a slurry of ceramic particles dispersed and dissolved within a liquid. The incorporation of the gaseous phase may be carried out by mechanical agitation, streaming gas, the gas given off by chemical reactions, and evaporation of the dissolvent [6]. The addition of active surface agents is a requisite to achieve the stability of the foamed structure [7] and proteins [8], and in some cases, gelation agents are also used for this purpose [9]. Recently, the use of natural organic compounds has been investigated to prepare stable ceramic foams, and several extracts of plants and fruits with outstanding foaming capacity have been identified [10].

Saponin is an organic molecule with valuable properties as a surfactant and foaming agent [11]; it is the main compound of the soap-nut fruit. The soap-nut contains ranges between 6% (nut) and 10% (pericarp) of saponins [12]. The saponin content in extracts is closely related to the extraction method and the soap-nut mass used. For example, 31.21% was obtained by a simple evaporative extraction (3.5 g/100 mL), while the use of a Soxhlet extractor allows a higher yield extraction with a saponin content of 87.23% (29.2 g/200 mL distilled water) [13]. The extract of the pericarp of this fruit is a biodegradable material, non-toxic and friendly to the environment [10]. For these reasons, it represents an excellent option in comparison to synthetic foaming agents employed in the fabrication of ceramic foams [14]. The use of this extract has been reported in the preparation of ceramic foams of Al_2_O_3_ [9,10], showing better interconnectivity than other natural products such as egg white [10]. Another ceramic of great interest is magnesium aluminate (MgAl_2_O_4_) spinel with a unique combination of mechanical and physical–chemical properties, such as high melting point, high mechanical strength at different temperatures, resistance to corrosion, low density, low coefficient of thermal expansion, and low thermal conductivity [15,16]. Foams of MgAl_2_O_4_ have been obtained by techniques including the sacrificial templates method using different freezing mediums of the pore-forming agents [17], a replica of polyurethane sponges [18], gel casting [19], and reactive sintering [20]. Despite the diversity of methods, all of them have some drawbacks, including processing steps at sub-zero temperatures, carbon residues after the pyrolysis process, and the use of neuro-toxic monomers. Inconveniently, the obtained foams have some closed cells [17,18,21]. Nonetheless, preparation of MgAl_2_O_4_ porous structures by the direct foaming technique using the extract of the soap-nut fruit has not been reported in the available literature to the best knowledge of the authors. 

This study seeks to fabricate stable and highly interconnected MgAl_2_O_4_ foams with different contents of ceramic, using the extract of the pericarp of the soap-nut fruit as foaming agent. The concentration of extract, time, and speed of stirring, the load of ceramic, and drying temperature were the parameters evaluated along with the type of agitator. The effects of these parameters were assessed in terms of foaming capacity, the stability of the foam, cell size distribution, and interconnectivity between cells. In subsequent work, the optimized porous structures, after sintering, will serve as preforms to manufacture by pressureless infiltration Al-based metal matrix composites reinforced with different contents of MgAl_2_O_4_.

## 2. Materials and Methods

### 2.1. Materials

A powder of MgAl_2_O_4_ with a D_80_ = 5.2 µm was used in this study to fabricate the ceramic foams. The chemical composition was quantified by X-ray fluorescence in a Bruker S8 Tiger equipment and was close to the composition reported by the provider (American Elements), as can be seen in Table 1. Darvan 7 (Vanderbilt Minerals) was used to disperse the ceramic particles and distilled water was employed as dissolvent. The extract of the pericarp of the soap-nut fruit was used as a foaming agent and was obtained following the procedure described in reference [10]. Briefly, the soap-nut fruit was broken to separate the shell from the seed. Moisture was removed by drying at 85 °C for 56 h, and then the pieces were powdered and finally sieved below 180 μm. Then, 50 g of pericarp powder was added into 1 L of distilled water and magnetically mixed at 1200 rpm for 3 h at 80 °C. Afterward, the solution was filtered through 11 μm pore size paper. The filtered extract was evaporated at 100 °C for 44 h, until a maroon color solid mass was obtained. Lastly, the solid was crushed into a fine powder and kept sealed at room temperature until use.

### 2.2. Characterization of the Soap-Nut Extract

The extract of the pericarp of the soap-nut fruit was characterized with a Bruker Tensor 27 Fourier transform infrared (FTIR) spectrometer. The sample was mixed with KBr in a 1:1000 ratio and compacted to be analyzed in the frequency range of 4000 to 400 cm^−1^ with a resolution of 4 cm^−1^.

The surface tension of aqueous solutions of pericarp extract was measured in the range of 0.001 to 3 wt.%. The measurements were carried out by the Wilhelmy plate method with a K20 KRÜSS force tensiometer at room temperature. The electrical conductivity was also measured for the same concentrations by employing a Hanna HI 2550 potentiometer with a HI76310 electrode. The measured values of surface tension and electric conductivity were used to obtain the critic micellar concentration.

### 2.3. Properties of the Foams

The viscosity of the slurries and ceramic foams was determined with a cone and plate rheometer. All measurements were performed employing 20 mL of suspension or foam at 25 °C, applying a shear rate of 10 s^−1^ for 1 min acquiring 60 measurements during the assay of each sample. For all slurries and ceramic foams prepared, the foaming capacity (*FC*) was calculated in percentage according to the following equation:

*FC* = [(*V_f_*/*V_i_*) − 1] × 100 (1)

where *V_f_* and *V_i_* are the final and initial volume, respectively.

### 2.4. Preparation of the Ceramic Foams

Different loads of 28, 37, 44, and 50 vol.% MgAl_2_O_4_ powder were added into 7 mL of distilled water followed by the addition of Darvan 7, 0.75 wt.% of the ceramic load. The suspension was blended with a magnetic stirrer at 500 rpm for 5 min. Then, different amounts of foaming agent, namely 0.5, 1, 3, and 5 wt.%, were incorporated into the suspension and mixed at 200 rpm for 10 min.

The ceramic slurry was directly foamed by stirring with a 4-arm impeller or with a disc agitator for stirring times between 1 to 4 min. The effect of the stirring rate was evaluated between 1070 to 1283 rpm and 1536 to 2613 rpm for the 4-arm and disc agitators, respectively. 

The ceramic foams were poured into rectangular aluminum molds with dimensions 88 × 12 × 7 mm and were dried at temperatures between 40 and 70 °C for 18 h. Finally, to confer some mechanical strength to the porous structures, the foams were partially sintered. This process was achieved by placing the foams into the hot zone of a tube furnace and heating to 1400 °C at 15 °C/min. The foams were held at temperature for 1 h under a high purity argon atmosphere, and then the furnace cooled naturally to ambient temperature. Sintering of the foams was carried out under an inert atmosphere of Ar in order to prevent any change in the surface chemistry of the porous structures; the inert atmosphere assisted the complete elimination of organic compounds from the foams by pyrolysis as white foams were obtained. Conversely, black foams were obtained when sintering in air.

### 2.5. Characterization of the Partially Sintered Foams

The morphology and size of the cells along with the interconnectivity between them were characterized by observing transverse sections of the partially sintered foams in the scanning electron microscope (SEM) JSM-6400 (Jeol Ltd., Tokio, Japan). The cell size distribution was analyzed, taking photomicrographs in an area of approximately 25 mm^2^ from the bottom to the top of the porous structures. Processing of the images was made in the computer using the software Sigma Scan Pro 5.

## 3. Results and Discussion

### 3.1. Characterization of the Soap-Nut Extract

**Figure 1:**
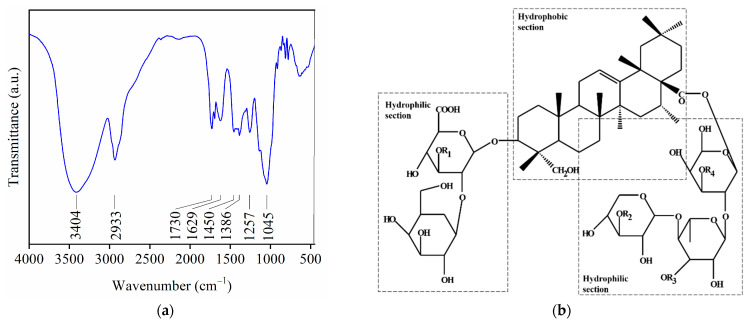
(**a**) FTIR spectrum of the soap-nut extract and (**b**) saponin structure.

Figure 1a shows the FTIR spectrum obtained for the soap-nut extract (SE). The spectrum presents a broad band of high intensity at 3404 cm^−1^ related to the tension of the O–H bond, corresponding to the hydroxyl groups in the SE. The band at 2933 cm^−1^ is assigned to the stretching, while the bands at 1450 and 1386 cm^−1^ are associated with in-plane bending of the C–H in methyl groups. The carboxylic groups are identified by the medium intensity bands at 1730, 1257, and 1045 cm^−1^, where the first corresponds to the stretching of C=O while the last two are related to the deformation vibrations of C–O. The band at 1629 cm^−1^ is ascribed to the stretching of the C=C bond. 

The aforementioned functional groups are characteristic of saponin, the main compound in the pericarp of the soap-nut, and have been reported for soap-nut extracts [9,13,14,22,23]. The structure of saponin (Figure 1b) has both hydrophobic and hydrophilic sections that contribute to the tensoactive properties. The hydrophobic or non-polar section named aglycone (triterpenoid) or sapogenin is linked to one or more monosaccharides by the glycosidic bond (hydrophilic section in the molecule) [13,24,25]. The FTIR spectrum confirms that saponin is present in the SE obtained in this work. The methodology followed to obtain the soap-nut extract allows reaching a yield of 36.16% for the indicated conditions.

**Figure 2:**
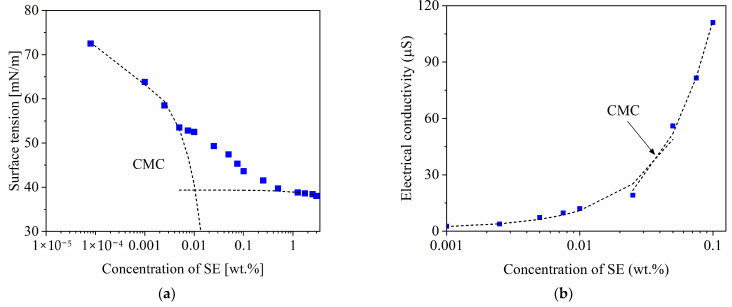
(**a**) Surface tension and (**b**) electrical conductivity at different content of SE at 25 °C.

Figure 2 displays the surface tension and the electric conductivity as a function of the SE concentration. An increase in the content of SE promotes a sharp diminution in the surface tension as can be seen in Figure 2a. This behavior is because the extract, mainly constituted by saponin molecules, has polar and non-polar groups that are adsorbed in the air–water interface, modifying the surface tension of the solution [13,23]. Afterward, the surface tension decreases to a lesser extent as the non-adsorbed molecules start segregating within the solution forming micelles [26]. This change was observed from the critical micellar concentration (CMC), 0.01 wt.%, as calculated with the surface tension data shown in Figure 2a. When the content of SE rises again, the surface tension remains practically constant at around 38 mN/m at 3 wt.% SE. This result is in agreement with the values reported in the literature by other authors [9,10,13,22,27], where the surface tension is between 32 and 40 mN/m when soap-nut extracts are used whilst the surface tension for commercial surfactants varies from 30 to 40 mN/m [27]. Thus, the soap-nut extract obtained exhibits similar behavior in comparison to commercial products.

In Figure 2b, a light increment is observed in the electrical conductivity of the distilled water when the concentration of SE augments; then, the curve describes a significant rise that corresponds to the formation of aggregates or micelles. From the plot of Figure 2b, a value of 0.038 wt.% was calculated for the CMC. According to the literature, the CMC values for the soap-nut extract as estimated from surface tension and electrical conductivity measurements are in the range between 0.01 to 0.05 wt.% [22,27,28]. According to Figure 2a, beyond the CMC value, the surface tension essentially remains constant at approximately 38 mN/m, corresponding to 3 wt.% SE. Taking into account that the foaming capacity is maximized at tensoactive concentrations higher than CMC [29], and based on the results obtained in this study, the soap-nut extract concentration was selected between 0.5 to 5 wt.% to assure enough tensoactives available to favor the preparation of stable foams.

### 3.2. Viscosity of the Slurries and Ceramic Foams

**Figure 3:**
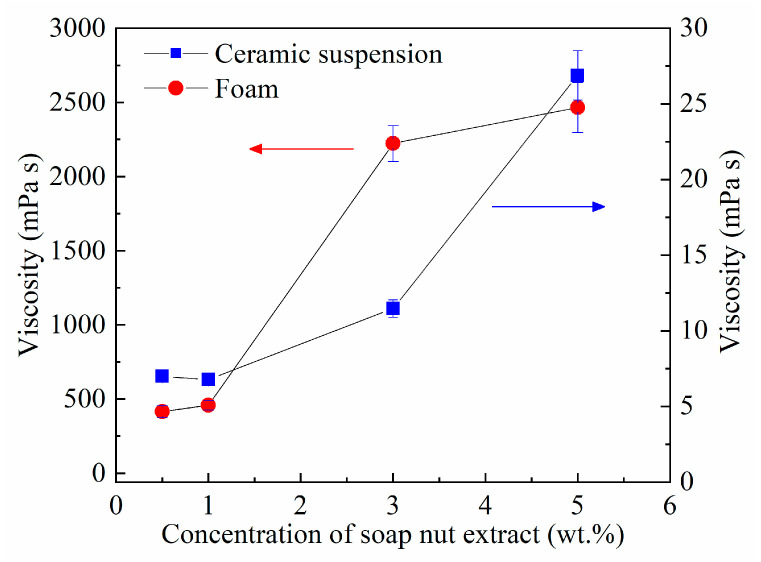
Viscosity of the ceramic suspensions and foams prepared with different content of SE at 25 °C, 37 vol.% MgAl_2_O_4_, and stirring at 1070 rpm for 2 min.

The results of viscosity measurements for the ceramic suspensions and foams as a function of the SE content are presented in Figure 3. The slurries were prepared using 37 vol.% MgAl_2_O_4_ and 0.75 wt.% Darvan 7, while the ceramic foams were obtained at the same composition and were foamed at 1070 rpm during 2 min.

Regarding the slurries, when the content of SE increases, a rise in viscosity from 7 ± 0.27 to 27 ± 1.64 mPa s was observed. The viscosity of the ceramic foams was noticeably higher than the slurries and increased with the content of extract from 415 ± 41 to 2466 ± 168 mPa s for 0.5 and 5 wt.% SE, respectively. The high viscosity of the ceramic foams is attributed to the air bubbles incorporated during the foaming process, besides the large number of molecules of saponin in the SE adsorbed at the gas-liquid interface [30].

### 3.3. Fabrication of the Ceramic Foams

#### 3.3.1. The Effect of the SE Content

**Figure 4:**
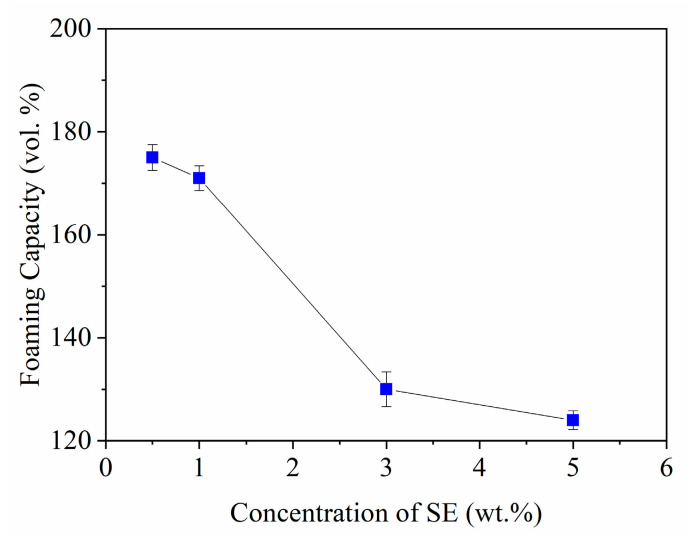
Foaming capacity of the ceramic slurries as function of SE content.

The results of the foaming trials of ceramic slurries with different content of SE are displayed in Figure 4 for a load of 37 vol.% MgAl_2_O_4_ and 0.75 wt.% Darvan 7. The foams were prepared by stirring at 1070 rpm using the 4-arm anchor impeller for 2 min. The foaming capacity was calculated with Equation (1). The results reveal that the maximum foaming capacity of 178 vol.% was obtained with 0.5 wt.% of SE. The presence of the SE leads to a reduction in the surface tension, resulting in low resistance to the formation of new interfaces by the mechanical force used.

The graph of Figure 4 shows that when the content of extract increases from 1 to 5 wt.%, the foaming capacity decreases from 174 to 125 vol.%, respectively. This is attributed to the rise in the viscosity, as seen in Figure 3, and to the loss in surface elasticity as a result of the strong packaging of the saponin molecules on the interface [25,31]. T-Gonzenbach and coworkers [6] observed similar behavior when foaming Al_2_O_3_ using carboxylic acids. The authors discovered that increasing the concentration of the foaming agent above the CMC results in a decrement of the foaming capacity. According to the authors, the high viscosity of the slurries does not allow the inclusion of more gaseous phase during foaming.

**Figure 5:**
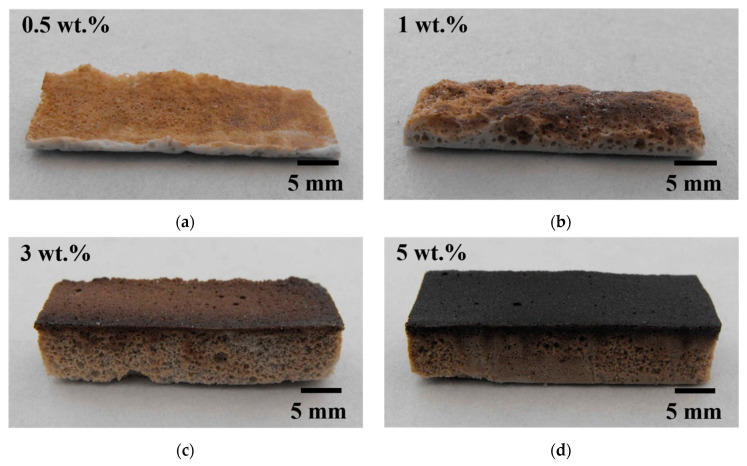
Effect of the content of SE on the macro characteristics of the MgAl_2_O_4_ foams. (**a**) 0.5 wt.%; (**b**) 1 wt.%; (**c**) 3 wt.%; and (**d**) 5 wt.%.

The ceramic foams obtained were dried at 60 °C for 18 h. Those prepared with 0.5 and 1 wt.% SE, in spite of exhibiting the greatest foaming capacity and minor viscosity, turned out to be unstable, as they completely collapsed during the first h of drying as shown in Figure 5. The content of the foaming agent does not provide enough saponin molecules to occupy the large air–water interface [9,32], as it is also distributed on the ceramic surface to enable foaming of the slurry [6]. In this scenario, thinning of the bubble films occur leading to rupture of the cells. Conversely, the foams prepared with 3 and 5 wt.% SE presented stability and proper resistance to be handled without inducing breakage after the drying process. Thus, these concentrations of SE in ceramic slurries allow forming a dense film around the air bubble, providing strength to deformation and standing local thinning [33].

**Figure 6:**
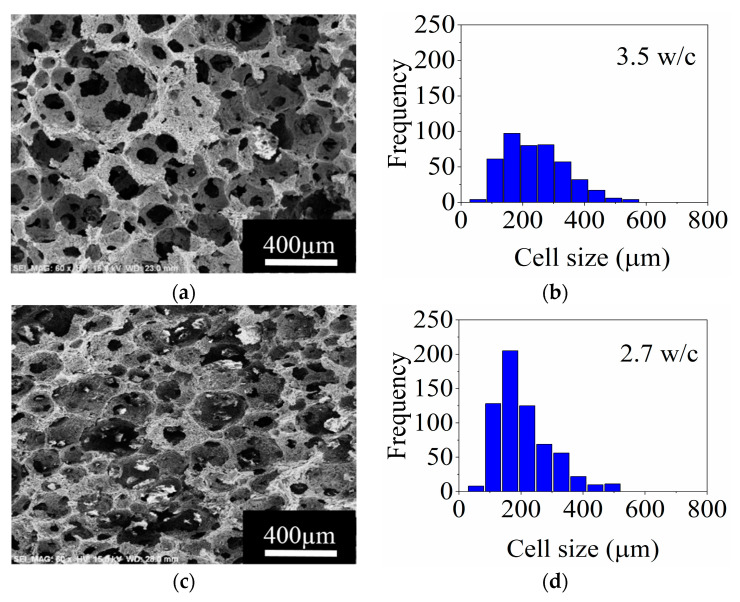
SEM micrographs and frequency histograms for the cell size of ceramic foams obtained using (**a**,**b**), 3 wt.% SE; and (**c**,**d**), 5 wt.% SE; respectively.

Figure 6 shows the SEM micrographs and the cell size distributions for the stable ceramic foams with 3 and 5 wt.% SE dried at 60 °C and sintered at 1400 °C. It is observed in Figure 6a,c that both porous structures are constituted by relatively spherical cells and circular windows. Generally speaking, the cell size of the foams decreases with the augment of the SE concentration in the ceramic slurries. The histogram of Figure 6b shows that a wide cell size distribution, between 55 and 550 μm, is obtained for the ceramic foam prepared with 3 wt.% SE. In the case of the ceramic foam obtained with 5 wt.% SE, the range in cell size reduced slightly, between 55 and 495 μm, showing a more homogenous and narrower distribution as seen in Figure 6d. The cell size that presented the higher frequency was 165 μm for both foams; nevertheless, the ceramic foams prepared with 5 wt.% SE presented greater frequencies.

On the other hand, the interconnectivity in ceramic foams is defined by the number of windows per cell (w/c). Based on the SEM micrographs, interconnectivities of 3.5 and 2.7 w/c were obtained for the ceramic foams produced with 3 and 5 wt.% SE, respectively (Figure 6b,d), indicating that a high concentration of SE in the foams reduces the interconnection between cells. This effect is triggered due to the low viscosity inducing thinning of the cell walls, and it is further favored during the evaporation of the solvent (water), since the viscoelastic behavior is lost at lower SE concentrations [9,34].

#### 3.3.2. The Effect of the Stirring Time

**Figure 7:**
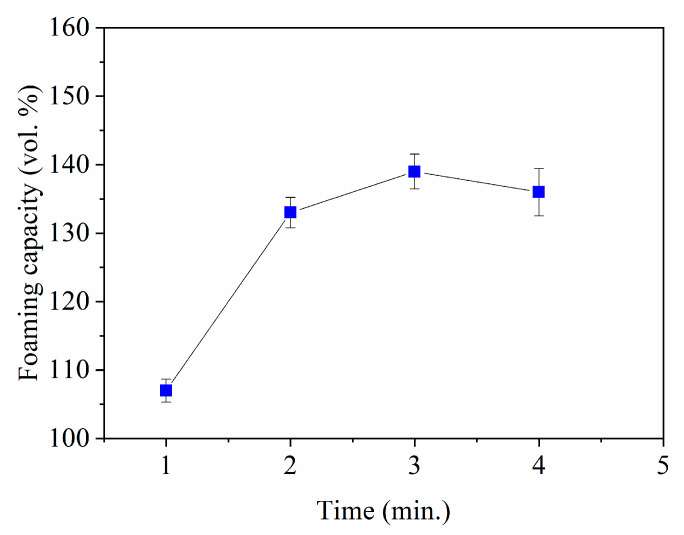
Foaming capacity as a function of stirring time for the slurries prepared with a load of 37 vol.% MgAl_2_O_4_ and 3 wt.% SE for a stirring rate of 1070 rpm.

The effect of the stirring time on the foaming capacity is presented in Figure 7 for the slurries prepared with 37 vol.% MgAl_2_O_4_, 0.75 wt.% Darvan 7, and 3 wt.% SE with the stirring rate maintained at 1070 rpm, conditions that allow the fabrication of stable foams without significant increase in viscosity. The foaming capacity exhibits an increase from 108% to 137% for stirring times of 1 and 3 min, respectively. For a longer stirring time (4 min), the foaming capacity remains virtually constant.

The foaming capacity changes with stirring time because stirring enhances the incorporation of air into the slurry while foaming and gives rise to redistribution of the saponin molecules on the interface [35]. After 3 min of stirring, the amount of air is maximized, and longer stirring times do not promote further foaming. 

**Figure 8:**
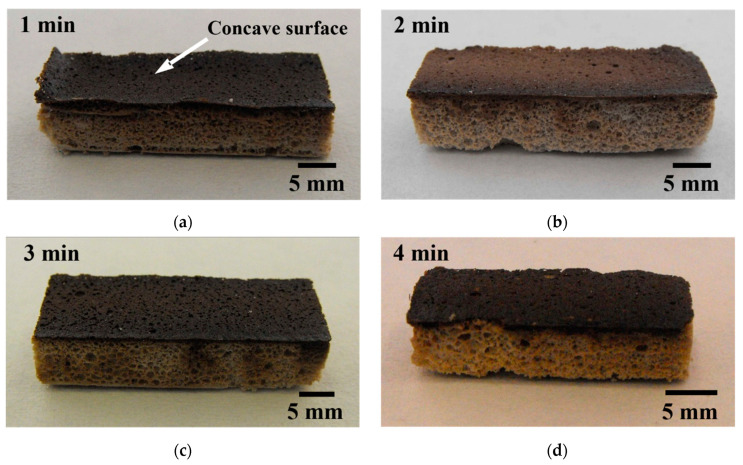
Effect of the stirring time on the macro characteristics of the MgAl_2_O_4_ foams. (**a**) 1 min; (**b**) 2 min; (**c**) 3 min; and (**d**) 4 min.

The foams produced by stirring the aqueous ceramic slurry for 1 min were found to be partially stable after drying at 60 °C for 18 h, as the top surface of the porous structure evidenced the formation of a concave curvature and some degree of delamination, as seen in Figure 8a. This defect is caused by the low viscosity, resulting in rupture of the cell walls induced by the drainage and a disproportionality effect [9,36]. The foams produced using longer stirring times were stable and did not show the concave curvature defect, as seen in Figure 8b–d.

**Figure 9:**
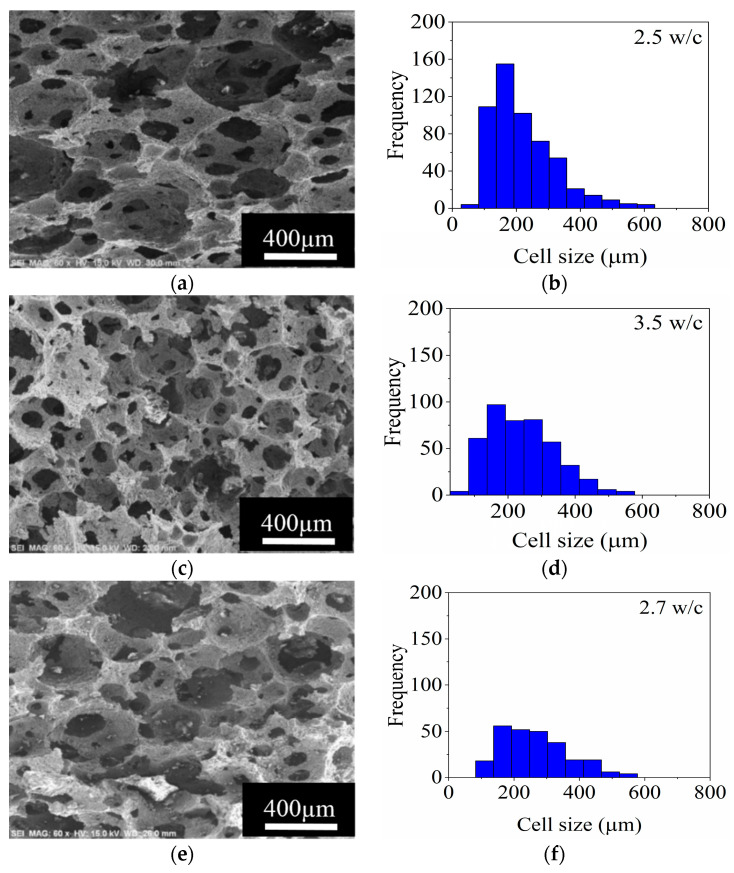

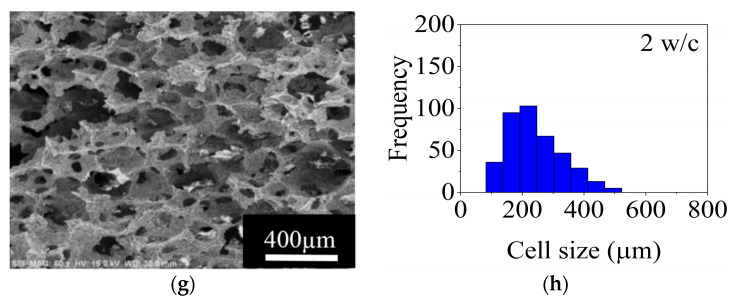
SEM micrographs and frequency histograms for the cell size of ceramic foams prepared with stirring times of: (**a**,**b**), 1 min; (**c**,**d**), 2 min; (**e**,**f**), 3 min; and (**g**,**h**), 4 min, respectively, with a load of 37 vol.% MgAl_2_O_4_ and 3 wt.% SE for a stirring rate of 1070 rpm.

The SEM micrographs and corresponding frequency histograms for the produced ceramic foams with different stirring times after drying at 60 °C are shown in Figure 9. The morphologies of the cells and windows of the ceramic foams are observed in Figure 9a,c,e,g. The features do not differ from those previously described in Section 3.3.1.

Cell sizes between 55 to 605 μm were measured for the ceramic foam obtained after 1 min of stirring. A slight diminution in the cell size range (55–550 μm) was registered for the ceramic foam stirred during 2 min. However, a more homogeneous distribution in cell size was achieved when the stirring time was 4 min. This improvement occurs because a longer time of agitation distributes the gaseous phase (air) more homogeneously in the ceramic foams, preventing the survival of large bubbles and producing a higher number of cells with similar sizes. The cell size of 165 μm has the higher frequency for stirring times between 1–3 min, while for the 4 min stirring foam, the maximum frequency was observed at 220 μm.

Regarding the effect of stirring time on interconnectivity, as the stirring time increased the interconnectivity between the cells considerably decreased so that some cells did not present the formation of windows. The maximum interconnection between cells 3.5 w/c was achieved after stirring for 2 min, while for times of 3 and 4 min, the w/c was 2.7 and 2, respectively, as seen in Figure 9.

Based on these results, stirring the slurry for 4 min allows the fabrication of ceramic foams with the higher frequency for the lower size cell, but to reach the maximum interconnection between cells, 2 min is enough.

#### 3.3.3. The Effect of Stirring Rate and Type of Impeller

The stirring rate is a very important variable in foam fabrication, as it dictates the cell size and the interconnection between cells. Thus, the effect of this parameter was evaluated in ceramic foams prepared with a load of 37 vol.% MgAl_2_O_4_, 0.75 wt.% Darvan 7, and 3 wt.% SE, using two types of impellers at different stirring rates for 2 min. Table 2 resumes the results of these trials in terms of the foaming capacity and stability of the foams.

In the first case, a 4-arm anchor impeller was used in the range between 1070 and 1283 rpm. The ceramic foams showed a similar foaming capacity in the interval of stirring rates employed, being 121 vol.% for 1240 rpm, while 133 vol.% was reached at 1070 rpm. This indicates that the 4-arm impeller promotes the inclusion of practically the same amount of gaseous phase (air) when the stirring rate increases. For all the trials, the foams were found to be stable.

Trials with a disc impeller seeking to enhance the foaming capacity were performed at different stirring rates and the results are also listed in Table 2. The results with this impeller revealed that the foaming capacity is considerably improved increasing the stirring rate. Thus, at 1536 rpm, the foaming capacity was 125 vol.%, whereas at 2613 rpm, an expansion of 268 vol.% was reached. The slurry foamed at 1536 rpm was partially stable due to the coalescence of bubbles during drying, since the SE content is not enough to maintain the gaseous phase integrated within the foam.

The increase in the foaming capacity arises owing to the high shear stress generated by the disc impeller by increasing the stirring rate [37]. In comparison with the 4-arm anchor impeller, the performance of the disc agitator was found to be more sensitive to the stirring rate and it is thus more efficient in creating new and numerous interfaces.

**Figure 10:**
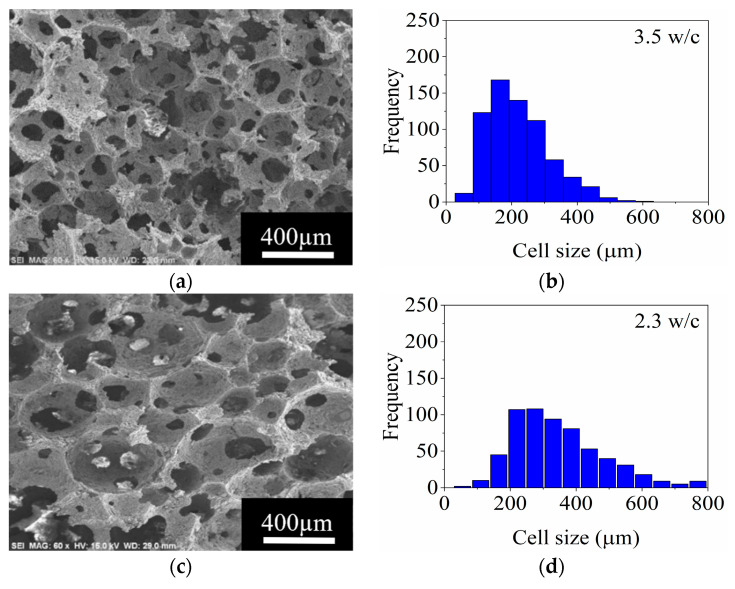

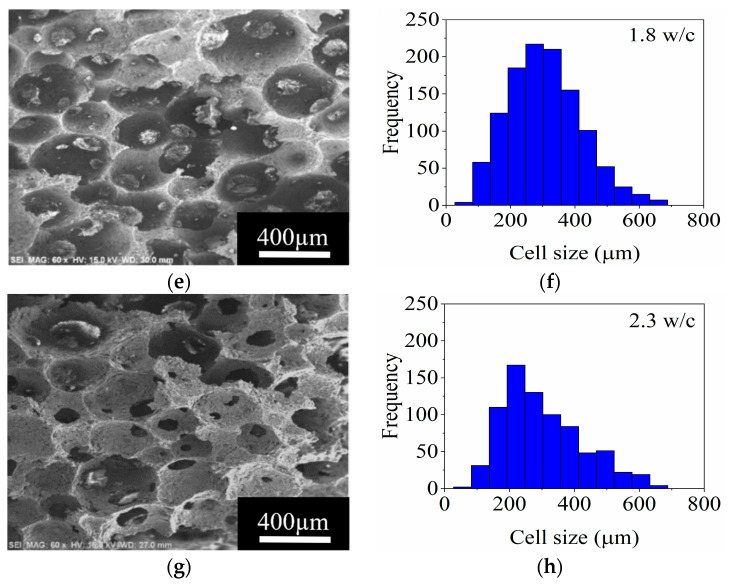
SEM micrographs and frequency histograms of the cell size obtained using the 4-arm anchor impeller at different stirring rates: (**a**,**b**), 1070 rpm; (**c**,**d**), 1194 rpm; (**e**,**f**), 1240 rpm; and (**g**,**h**), 1283 rpm, respectively.

**Figure 11:**
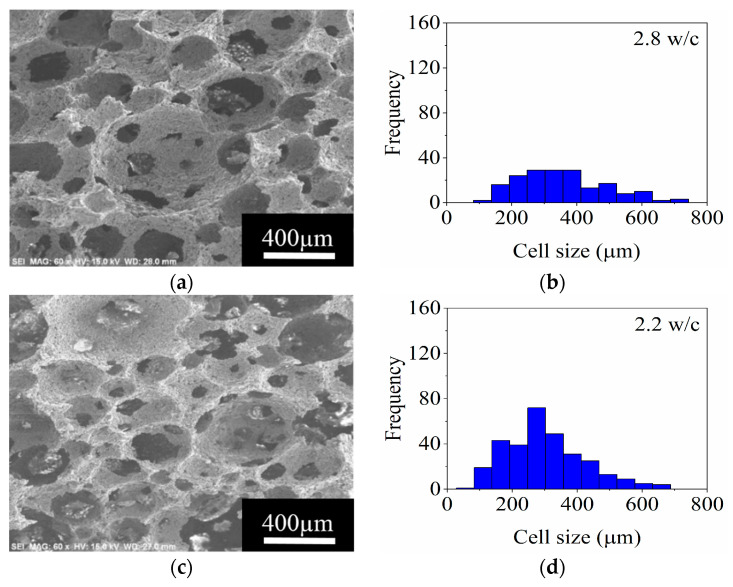

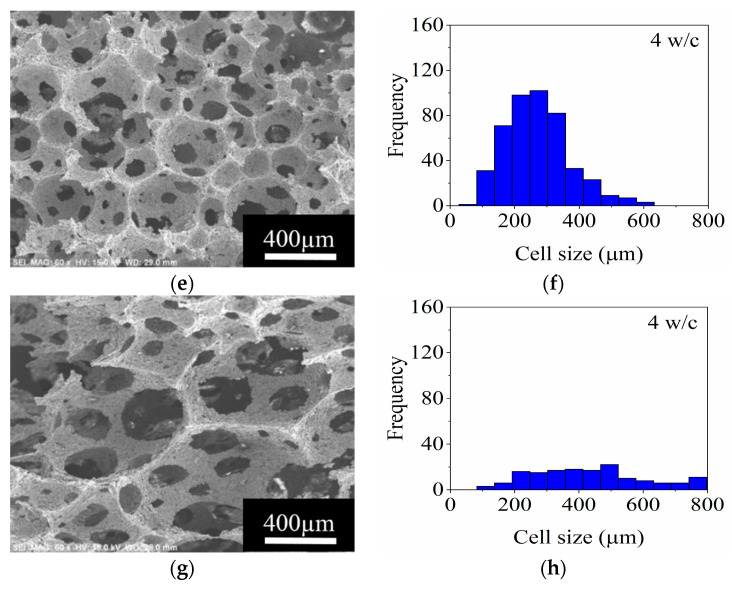
SEM micrographs and frequency histograms of the cell size obtained using the disc impeller at different stirring rates: 1536 rpm, (**a**,**b**); 2120 rpm, (**c**,**d**); 2360 rpm, (**e**,**f**); 2613 rpm, and (**g**,**h**); respectively.

In order to evaluate the cell size and interconnectivity between cells, the fabricated ceramic foams were observed and imaged in the SEM. Figure 10 and Figure 11 exhibit the characteristic porous structures obtained by stirring the ceramic slurries with the 4-arm anchor and disc impeller, respectively, along with their corresponding frequency histograms.

The characterization of the foams shows that the 4-arm impeller produces porous structures with a large frequency in the cell size of 165 µm with a stirring rate of 1070 rpm. When the stirring rate is increased to 1194 and 1240 rpm, the highest frequency cell size is 275 µm for both cases. A decrement in the cell size occurs when stirring at 1283 rpm. This behavior is ascribed to the fact that, at this stirring rate, the foaming capacity is maximum, and consequently, thinner films are generated. A greater number of small bubbles generates a higher gas pressure than larger bubbles according to the Laplace equation [38,39]. In this scenario, a small air bubble diffuses through the water layer to a higher bubble increasing its size. The smaller bubbles shrink and eventually disappear, widening the cell size distribution of the foam [6,38]. Regarding the interconnectivity between cells, the measured values show a decrement due to disproportionality effect and an increase in the viscosity, 3.5 w/c was obtained at 1070 rpm whilst 2.3 w/c was estimated for 1283 rpm, as seen in Figure 10.

In the counterpart, the results of the ceramic foams prepared using the disc impeller showed a reduction in the cell size as the stirring rate increased from 1536 to 2360 rpm as observed in Figure 11. A wide cell size distribution and a larger size are obtained when stirring at 2613 rpm.

It is notable in the frequency histograms a reduction in the cell size in addition to an increase in the frequency of 275 μm cell size. This is related to the generation of cells with a uniform size with the stirring rate used. At 2613 rpm, the coalescence of bubbles to generate others of greater size is promoted by the elevated stirring rate, reflecting an increment in cell size.

The interconnection between cells raises from 2.8 to 4 w/c using the disc impeller by increasing the stirring rate. This effect is opposite to that observed for the 4-arm anchor impeller. Therefore, for the preparation of ceramic foams with different ceramic loads, the disc impeller was chosen.

#### 3.3.4. The Effect of the MgAl_2_O_4_ Content

**Figure 12:**
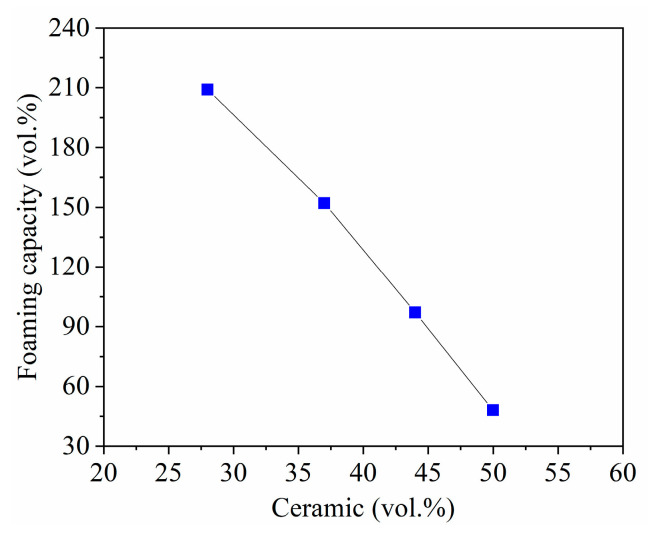
Foaming capacity of the MgAl_2_O_4_ slurries at different ceramic loads.

Figure 12 displays the foaming capacity of the slurries stirred with the disc impeller as a function of MgAl_2_O_4_ load. A significant decrease is observed in the foaming capacity when the ceramic load augments. The ceramic slurry containing 28 vol.% MgAl_2_O_4_ reached an expansion of 209 vol.%, while that prepared with 50 vol.% MgAl_2_O_4_ showed only 48 vol.%. At these loads of ceramic in the slurries, the ceramic content in the foams corresponds to 7, 11, 21, and 30 vol.% MgAl_2_O_4_, respectively, as measured after partial sintering of the porous bodies, using Archimedes’ principle. The fabricated foams were stable after drying except those prepared with 50 vol.%, in which collapse was observed.

**Figure 13:**
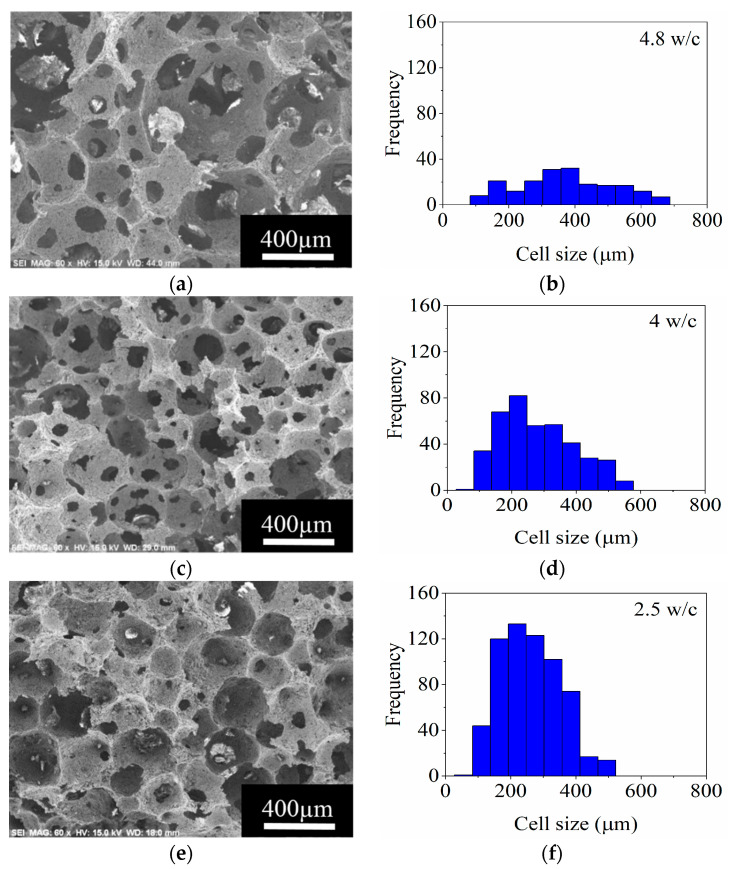
SEM micrographs and frequency histograms of cell size for ceramic foams prepared with (**a**,**b**), 7 vol.%; (**c**,**d**), 11 vol.%; and (**e**,**f**), 21 vol.% MgAl_2_O_4_, respectively.

The micrographs of the foams obtained with 7, 11, and 21 vol.% MgAl_2_O_4_ at 2360 rpm are shown in Figure 13. For all the foams, the interconnected cell microstructure is maintained, and an increase in ceramic load induces a reduction in the size of the cells.

The 7 vol.% MgAl_2_O_4_ foam shows zones with large cell size and thereby presents a wide cell size distribution, from 100 to 600 μm, where 385 μm is the cell size of major frequency (Figure 13a). A more homogeneous cell size distribution (55–550 μm) was obtained for the ceramic foam prepared with 11 vol.% MgAl_2_O_4_ and 220 μm corresponds to the maximum frequency as appreciated in Figure 13d.

The foam with 21 vol.% MgAl_2_O_4_ was characterized with a range of cell size between 100 to 495 μm. The diminution in cell size is attributed to the low foaming capacity with the load of ceramic as a consequence of the lack of ability to incorporate the gaseous phase (air) into the ceramic slurry while foaming. The interconnection between cells diminished when the MgAl_2_O_4_ load increased. For instance, the fabricated ceramic foams with 7 and 21 vol.% MgAl_2_O_4_ showed an interconnectivity of 4.8 and 2.5 w/c, respectively (Figure 13a–f). Therefore, the interconnection between cells is closely related to the ceramic content.

#### 3.3.5. The Effect of the Drying Temperature

**Figure 14:**
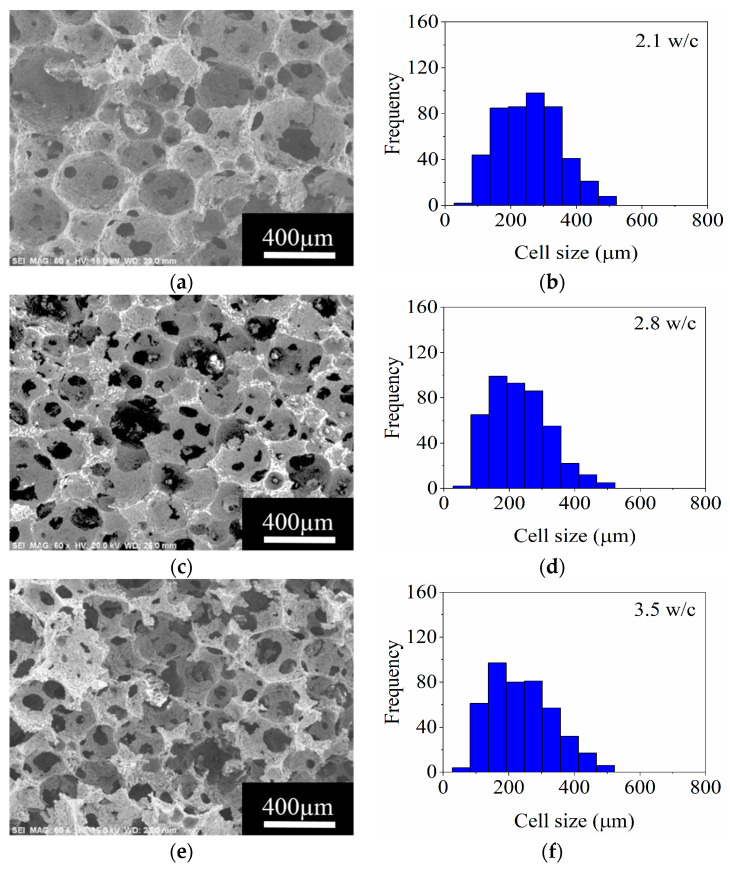

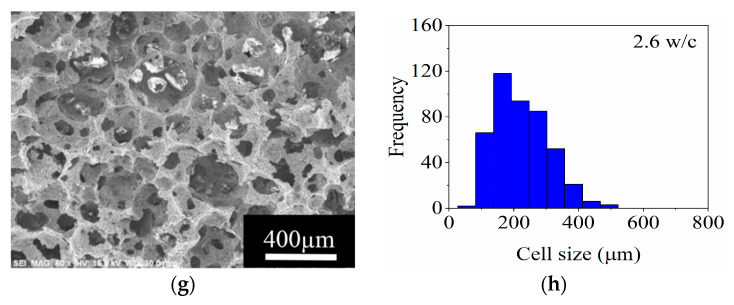
SEM micrographs and frequency histograms of cell size for the ceramic foams obtained after drying at: (**a**,**b**), 40 °C; (**c**,**d**), 50 °C; (**e**,**f**), 60 °C; and (**g**,**h**), 70 °C, respectively.

The stability, cell size, and interconnectivity between cells in the fabricated ceramic foams can be influenced by the drying temperature. So, this parameter was evaluated in the range of 40–70 °C for foams prepared with a load of 37 vol.% MgAl_2_O_4_ in the aqueous ceramic slurry and 0.75 wt.% Darvan 7. The morphology and the cell size distribution are shown in Figure 14, and the interconnection between cells is evident in all cases in the SEM micrographs. In Figure 14a, it is observed that the size of the cells is greater for the foam dried at 40 °C. That is because the temperature used for removing the solvent is low, allowing some level of drainage and causing coalescence of some cells. The foams dried at 40 and 50 °C were partially stable, drainage occurs before complete drying of the porous structures, and shrinkage of the bodies is evident. Conversely, a volume expansion was seen in the top section of the ceramic foam promoted by the rapid removal of the solvent when drying at 70 °C. Under these conditions, a large amount of gaseous phase is released, augmenting the internal pressure and thus inducing the volume expansion. Nevertheless, after drying, a completely stable ceramic foam without defects was obtained at 60 °C. At this temperature, the abrupt drainage of the solvent and further gas released are avoided [9].

The data shown in Figure 14a–h reveal that the interconnectivity between cells is improved as the temperature increases. The formation of windows between cells is low at 40 °C, 2.1 w/c. This characteristic, however, increases to a maximum at 60 °C with 3.5 w/c, whereas, when drying at 70 °C, there is a decrease to 2.6 w/c. Thus, it is concluded the drying temperature of 60 °C is adequate for obtaining stable foams with high interconnectivity. Pradhan and Bhargava [9] reported delamination defects, transversal cracks, and collapse in foams fabricated with soap-nut extract and a load of 35 vol.% Al_2_O_3_ after drying between 30 to 60 °C. Nevertheless, the results of this study disclosed a window for fabrication of MgAl_2_O_4_ ceramic foams without defects.

#### 3.3.6. Foaming Mechanism by the Soap-Nut Extract

**Figure 15:**
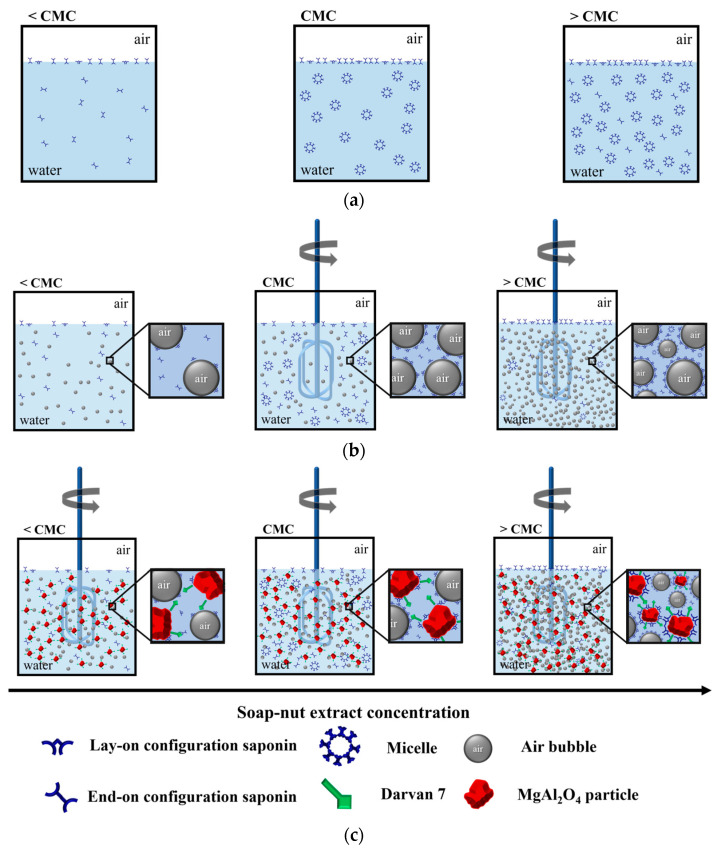
Schematic of the interaction mechanisms of soap-nut extract: (**a**) in water solution, (**b**) during foaming in water, and (**c**) during foaming the ceramic slurry.

To explain the foaming mechanism by the soap-nut extract, Figure 15 illustrates a schematic of its behavior in water solution during foaming and in the ceramic foam fabrication at different concentrations of the extract (<CMC, CMC, >CMC). As previously stated, the soap-nut extract contains saponin with outstanding foaming properties, and Figure 1b shows the hydrophilic and hydrophobic sections of saponin. The saponin can be oriented in both “lay-on” or “end-on” configurations, where two hydrophilic sections or only one, respectively, are arranged toward water [25]. Thus, in Figure 15 these configurations were considered. The soap-nut extract in water at low concentration (<CMC) promotes a diminution in surface tension and electrical conductivity; the saponin from the soap-nut extract was distributed in the bulk solution in “end-on” configuration, and it was arranged at the water–air interface with the hydrophilic section toward the aqueous phase. When the soap-nut extract concentration corresponds to the CMC, the saponin is mainly in micellar arrangement, and the water–air interface saturates (Figure 15a). In this condition, the surface tension and electrical conductivity dramatically diminished. If the concentration exceeds the CMC, micelles and tensoactive molecules are distributed in bulk solution, the interface is completely saturated by the tensoactive, and the physicochemical properties are not substantially modified.

During foaming (Figure 15b), the inclusion of air bubbles is promoted by the impeller. The saponin that, at the beginning, was distributed in the bulk solution, is now arranged at the water–air interface and distributed around the air bubbles stabilizing both phases in the system. Because the soap-nut extract content is low (<CMC), these foams tend to be unstable, and coalescence spontaneously occurs. When the soap-nut extract corresponds to the CMC, reduction in the surface tension results in low resistance to the formation of new interfaces by the mechanical force used. The micelles are destroyed by the agitation, and the saponin molecules are distributed on the newly created interfaces, enabling the largest incorporation of gaseous phase (air) and thereby the maximum foaming capacity. A high concentration of soap-nut extract facilitates the role of saponin to stabilize the porous structure with the air added. 

The MgAl_2_O_4_ is hydrophobic, and fabrication of ceramic foam requires a dispersant such as Darvan 7 (sodium polymethacrylate) that was added in the constant proportion of 0.75 wt.% to the ceramic. Darvan 7 is absorbed in the surface of the MgAl_2_O_4_ particles, stabilizing the suspension by electrostatic attractions [40], and during foaming of the ceramic suspension, the saponin is also adsorbed at the MgAl_2_O_4_ surface [6], as illustrated in Figure 15c. However, Darvan 7 acts as a destabilizer of the foam, as it prevents absorption of the saponin molecules at the solid–liquid interface [38,39]. In consequence, the amount of soap-nut extract must be high enough (≥CMC) to be arranged at the water–air interface and modify the MgAl_2_O_4_ surface to generate stable foams and promote high foaming capacity.

Finally, once the ceramic foam was prepared, it was set for the drying process. During the water evaporation, the thinning of the cell walls is promoted by the loss of viscoelastic behavior. The air pressure generated on the cells is higher than the interfacial elasticity limit of the film [32,34]. Consequently, the bubbles break giving rise to the formation of interconnected cells and the opened porous microstructure is achieved.

## 4. Conclusions

The use of the soap-nut pericarp extract as a foaming agent allows the fabrication of stable MgAl_2_O_4_ foams without other additives due to its ability to stabilize the porous ceramic structure. The evaluation of the parameters that affect the system showed that stable foams, in the range of 7 to 21 vol.% MgAl_2_O_4_, may be prepared with 3 wt.% SE, stirring for 2 min at 1070 and 2120 rpm for the 4-arm anchor and disc impeller, respectively, and drying of the porous structure at 60 °C. Observation of the fabricated MgAl_2_O_4_ ceramic foams exhibited a high interconnection between cells with relatively spherical cell morphologies and highly circular windows without defects. The modification of these parameters may significantly change the foaming capacity and cell sizes. Further foaming trials need to be done to manufacture foams with a greater vol.% of MgAl_2_O_4_.

## Figures and Tables

**Table 1:** Chemical composition of the MgAl_2_O_4_ powder (wt.%).

	TiO_2_	MgO	SiO_2_	Na_2_O	Fe_2_O_3_	K_2_O	CaO	Al_2_O_3_	Other Oxides
Provider	0.02	24.19	0.14	0.24	0.08	0.02	0.24	75.07	-
XRF	N.D. ^1^	22.7	0.292	N.D. ^1^	0.0871	N.D. ^1^	0.457	76.4	0.06

^1^ Not detected.

**Table 2:** Influence of the type of impeller and stirring rate on the foaming capacity and stability of the foams prepared with a load of 37 vol.% MgAl_2_O_4_ and stirring for 2 min.

Type of Impeller	Stirring Rate [rpm]	Foaming Capacity [vol.%]	Stability
4-arm anchor	1070	133	Stable
	1194	129	Stable
	1240	121	Stable
	1283	131	Stable
Disc	1536	125	Partially stable
	2120	132	Stable
	2360	152	Stable
	2613	268	Stable

## Data Availability

Data is contained within the manuscript.
